# Disability-friendly healthcare at public health facilities in Bangladesh: a mixed-method study to explore the existing situation

**DOI:** 10.1186/s12913-022-08538-6

**Published:** 2022-09-20

**Authors:** Nawshin Torsha, Farah Naz Rahman, Md Shafkat Hossain, Hasina Akhter Chowdhury, Minjoon Kim, S. M. Mustafizur Rahman, A. K. M. Fazlur Rahman, Aminur Rahman

**Affiliations:** 1 Centre for Injury Prevention and Research, Bangladesh (CIPRB), House: B120, Road: 07, New DOHS, Mohakhali, 1206 Dhaka, Bangladesh; 2 United Nations Children’s Fund (UNICEF), UNICEF house, Plot E#30, Syed Mahbub Morshed Avenue, Sher-E-Bangla Nagar, Agargaon, 1207 Dhaka, Bangladesh; 3 Non-communicable Diseases Control Program, Directorate General of Health Services (DGHS), Mohakhali, 1212 Dhaka, Bangladesh

**Keywords:** Disability friendly healthcare, Bangladesh, Public healthcare facilities, Health service, People or persons with disability

## Abstract

**Background:**

Several strategies and policies are being implemented in Bangladesh to address the healthcare needs of people with disabilities, who comprise about 10% of the country’s total population. However, these measures are not adequate to provide accessible or friendly healthcare to the people with disabilities. This study aimed to explore the disability-friendliness of healthcare facilities, and the challenges of people with disabilities in terms of access to 1) information and communication, 2) access to infrastructure, and 3) providers’ capacity in Bangladesh.

**Methods:**

An explanatory sequential mixed-method study was conducted, including a cross-sectional survey of healthcare facilities, followed by structured-interview with people with disabilities and healthcare managers, and qualitative interviews among people with disabilities or their caregivers, healthcare providers (HCPs), policymakers, and community leaders. Data were collected from 150 public healthcare (primary-to-tertiary) facilities and from 300 people with disabilities in 16 districts across Bangladesh between January-December 2019. An observational checklist and structured questionnaires were used to assess the situation of healthcare facilities, and literature-guided guidelines were used for qualitative interviews. During analysis, the disability-friendliness of healthcare facilities were quantified through a scoring system, and thematic analysis of qualitative data was performed to identify the challenges of implementing disability-friendly healthcare (DFHC).

**Results:**

The score for providing DFHC was low across all the four objectives in the healthcare facilities. The highest score (mean percentage) was observed in the infrastructure domain: 29.3 ± 20.5, followed by communication: 18.2 ± 4.8, and information: 14.6 ± 6.22, and the lowest (0.93 ± 7.1) score was for capacity of the HCPs to provide DFHC. Mean percentage scores for access to 13 infrastructure points were low, and extremely low scores were found in areas such as access to elevators (5.6 ± 5.0), ticket counters (7.3 ± 17.7) and toilets (10.6 ± 9.3). Furthermore, about 59.1% of people with disabilities expressed dissatisfaction regarding access to information and communication. The majority (98.2%) recommended that training of HCPs can improve the situation.

**Conclusion:**

This study revealed that most of the public health facilities in Bangladesh were not disability-friendly. Findings can inform development of a national disability-friendly policy with implementation guidelines.

**Supplementary Information:**

The online version contains supplementary material available at 10.1186/s12913-022-08538-6.

## Background

People with disabilities comprise 10% [1] of Bangladesh’s total population according to a survey conducted by Centre for Disability in Development (CDD), and have disproportionately higher healthcare needs [2]. “Disability refers to a situation where any person who is affected with long-term or permanent physical, psychological, intellectual, developmental and sensory-related damages or obstacles which in interaction with attitudinal and environmental barriers toward the person restricts the person’s equal and effective participation in society”. This translation of the definition has been used in the document “Defining Disability: A Guideline for Medical Doctors and Primary Health Care Workforce” developed by the government of Bangladesh. Several comprehensive strategies and strong policies are implemented by countries around the world to address the needs of people with disabilities [3, 4]. Disability inclusive health services is such a strategy that aims to ensure adequate health services for persons with disabilities and to reinforce the United Nations Convention on the Rights of Persons with Disabilities (UNCRPD), 2006 [5].

**Fig. 1:**
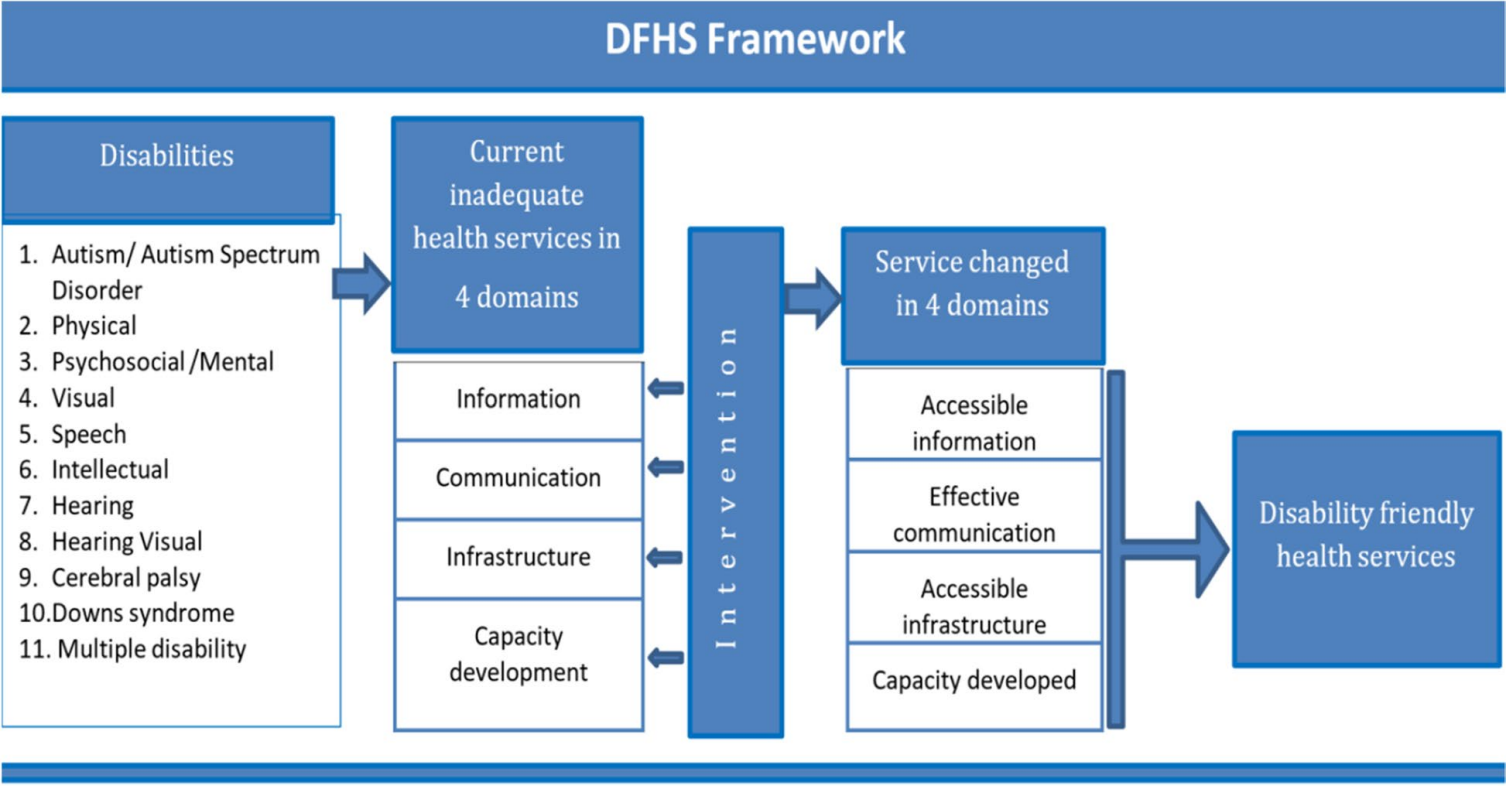
A conceptual framework highlighting the major domains for Disability Friendly Health Services (DFHS) in Bangladesh

Following the UNCRPD declaration, several countries have emphasized integrating disability rights into their health system. The government of Ireland developed national guidelines to promote inclusive healthcare services, which outlined the provision of accessible information, communication and hospital services for persons with disabilities [6]. Along similar lines, the Malaysian government implemented the ‘Global Disability Plan of Action,’ which focused on capacity development of healthcare professionals in addition to accessibility to health facilities and services [7]. The United Nations Division for Social Policy Development has also developed a toolkit on disability for the African continent to establish inclusive health services for persons with disabilities. This toolkit sets out actions to address barriers to healthcare for people with disabilities, which includes promoting universal infrastructure and disability-inclusive health information to reduce physical, communication and information barriers [8]. To summarize, access to information and communication, access to health facility infrastructure and capacity development of healthcare staff are the major domains that have emerged from various research and programmes from different countries for establishing disability friendly healthcare. Based on the findings of these studies from around the world, it has been hypothesized that challenges and barriers in these major domains must be identified, and targeted interventions should be developed, in order to establish disability-friendly healthcare services in Bangladesh. Figure 1 presents a conceptual framework has been developed based on this hypothesis.

While several countries have taken steps to establish disability-inclusive health services, South Asian countries still lack strategies to ensure adequate access to health services for persons with disabilities, despite having a considerable burden [9]. Due to various socio-economic and cultural differences, the strategies of high-income countries may not be applicable in this region. The strategy for establishing disability friendly healthcare must be country- and context-specific. Bangladesh is a signatory participant of UNCRPD [10], and disability and the rights of persons of with disabilities have been considered in different national policies, plans and programmes. ‘The Rights and Protection of Persons with Disabilities Act, 2013’ defines disability, mentions 11 types of disability and emphasizes the rights of persons with disabilities in line with the principles of UNCRPD [11].

Although the government of Bangladesh has enacted some policies and plans acknowledging disability issues, there is no action in place to provide disability friendly healthcare. Bangladesh is committed to providing ‘Good health and wellbeing for all’ under Sustainable Development Goal-3, and ensure appropriate care to persons with disabilities will help the country meet this goal as well as achieve universal health coverage. This study, therefore, explored the current situation of healthcare delivery systems in Bangladesh for providing disability friendly healthcare and identified the challenges in terms of access to information and communication, access to infrastructure and capacity of healthcare providers.

## Methods

### Design, settings, and population

The study utilized an explanatory sequential mixed method approach. The quantitative component included a cross-sectional survey that assessed all tiers of healthcare facilities in Bangladesh across four domains: information, communication, infrastructure and capacity of HCPs. Moreover, Focus Group Discussions (FGDs), Key Informant Interviews (KIIs) and In-depth Interviews (IDIs) were carried out among health policy makers, health care providers (HCPs) and administrators, disability related non-government organization (NGO) workers, community leaders, and people with disabilities including women, children and their caregivers, as a part of the qualitative component of the study. Following inclusion and exclusion criteria was considered for this study:

- Inclusion Criteria:
  i Government healthcare facilities of all tiers (primary, secondary, tertiary) were considered in this study for facility assessment survey.
  ii Persons with disabilities aged ≥ 16 years who had sought healthcare in last 5 years were considered in this study.
- Exclusion Criteria:
  i Private and NGO-based healthcare facilities were excluded.
  ii Persons with disabilities aged less than 16 years were excluded.
  iii Persons with disabilities who hadn’t sought healthcare in health facilities in the last 5 years were excluded

### Sample size & sampling strategy

**Fig. 2:**
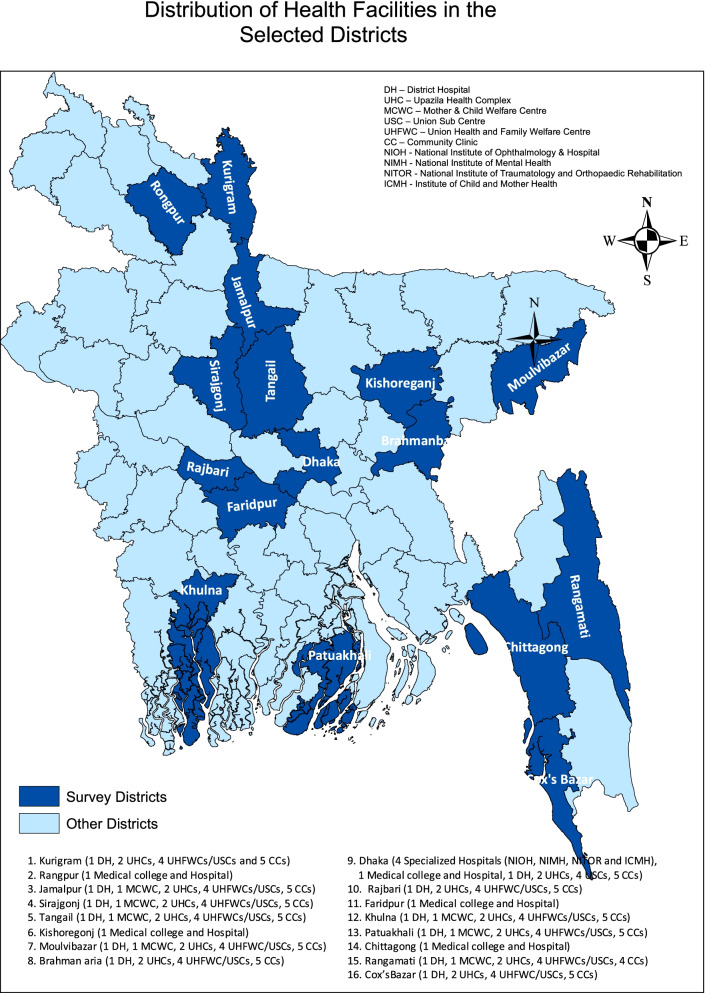
Map of the health facilities included in the study across different regions in Bangladesh

A total of 150 public healthcare facilities were selected randomly from 16 selected districts in Bangladesh from a government health facilities list (BDHS 2014), with facility level stratification. Among the 150 selected public health facilities, there were nine tertiary-level health facilities (four specialized hospitals and five medical college hospitals), 18 secondary-level health facilities (12 district hospitals and six Maternal and Child Welfare Centers (MCWCs)), and 123 primary-level health facilities (24 Upazila health complexes (UHCs), 50 Union Health & Family Welfare Centers (UHFWCs) and Union Sub-centres (USC), and 49 Community Clinics). The health administrator from each health facility was selected for qualitative interview. Figure 2 depicts the map of health facilities in various regions of Bangladesh that were included in the study.

A total of 300 people with disabilities, two from each facility or adjacent community (one man and one woman), were selected to explore their access and barriers to health facilities and obtain their suggestions for improvement. If any persons with disabilities was available at the health facility, an exit interview was conducted and then the snowball technique was applied to identify the persons with disabilities and their caregivers for focus group discussion. However, during health facility visits if no person was found, the interviewers took help from the health administrators to identify the people with disabilities who had recently received treatment from the facility. In cases where persons with disabilities were unable to provide interview, interview was conducted with self-reported primary caregiver and written consent was obtained from them. Furthermore, we purposively selected participants for 11 KIIs, 63 IDIs, and 12 FGDs [with 6–8 participants in each group].

### Research instruments

An observational checklist was adapted from the Ireland ‘National Guidelines on Accessible Health and Social Care Services’, and Malaysian and UN Toolkit of Disability-inclusive Health Services in Africa [8]. The checklist has four previously mentioned domains and specific indicators under each domain to assess a health facility’s status in a certain domain on accessibility for people with disabilities. The responses to the indicators were categorized as ‘Yes/No’, implying the presence or absence of a service for people with disabilities, and was determined by HCPs and researchers.

In addition, literature guided research instruments were developed with (10- 14) key questions focusing on the four domains for KIIs with key stakeholders, IDIs, and FGDs with service providers and people with disabilities, as well as a structured-questionnaire for health mangers of public facilities. The qualitative guidelines were designed with themes and probes to elicit the above-mentioned respondents’ perceptions and recommendations on service accessibility and barriers in the four key domains. Following expert consultation and field testing, the instruments were finalized.

### Data collection procedure

Four Research Assistants (RA) with medical backgrounds were trained to assess accessibility of health facility infrastructure through the observation checklist, and to interview the facility administrators and people with disabilities using structured questionnaires.

A team of one RA and one anthropologist conducted each KII and IDI. The RA interviewed the respondents while the anthropologist took notes and facilitated the audio-recording of the session (with the respondent’s permission) for subsequent analysis. To conduct each FGD, one RA and two anthropologists were engaged. One of the anthropologists facilitated the discussion, the other one was engaged in note taking and recording, and the RA was responsible for the overall management of the discussions.

### Data analysis

Descriptive analysis of quantitative data was conducted to assess the infrastructure status of health facilities, and access to information and communication for people with disabilities. All analyses were performed using SPSS Version 23.0. We measured our three outcomes of interests in the following way:

- Infrastructure status of health facilities (Table 1)

**Table 1:** Mean percentage score of different service points of health facilities infrastructures in different level

**Disability friendly infrastructure and service indicators**	**All Facilities** **(** ***n*** ** = 150)**	**Tertiary level healthcare (** ***n*** ** = 9)**	**Secondary level healthcare (** ***n*** ** = 17)**	**Primary level healthcare (** ***n*** ** = 124)**
Entrance	38.7 ± 25.3	55.6 ± 16.6	49.0 ± 23.9	36.0 ± 25.3
Staircase	20.4 ± 31.8	77.8 ± 21.0	47.1 ± 25.4	12.6 ± 26.6
Ramp (where applicable)	15.7 ± 25.9	55.6 ± 29.6	32.9 ± 31.5	10.5 ± 21.1
Reception and waiting areas	19.9 ± 18.6	46.0 ± 13.8	27.7 ± 19.8	16.9 ± 17.0
General Areas and circulation	33.8 ± 24.6	68.9 ± 17.6	37.6 ± 23.3	30.8 ± 23.3
Toilet facilities	10.6 ± 9.3	9.8 ± 11.7	5.2 ± 5.7	11.4 ± 9.3
Consulting rooms	44.0 ± 34.2	66.7 ± 25.0	61.7 ± 37.6	39.9 ± 33.1
Hospital wards	12.5 ± 22.0	41.6 ± 17.6	35.3 ± 21.7	7.3 ± 17.9
Out-patient department	12.4 ± 20.6	44.4 ± 16.6	31.4 ± 14.3	7.5 ± 17.9
Signage	27.3 ± 23.3	40.3 ± 22.3	21.3 ± 17.5	27.1 ± 23.7
Elevators	5.6 ± 5.0	63.9 ± 39.7	16.2 ± 33.0	-
Medicine corner/Pharmacy	18.2 ± 24.1	16.7 ± 25.0	26.5 ± 25.7	17.3 ± 23.8
Ticket counter	7.3 ± 17.7	11.1 ± 22.0	17.6 ± 24.6	5.6 ± 15.8
Total mean percentage score	**20 ± 13.7**	**46.0 ± 10.3**	**31.5 ± 8.9**	**17.1 ± 11.6**

[Results are expressed as mean percentage and 1 standard deviation]

The data collected from the observation checklist, which had 13 infrastructure indicators, was used to assess the infrastructure status. Each indicator had sub-points for which a single score (1) was assigned. We calculated the mean score of each indicator (sum of sub points for each infrastructure/total number of sub points), as well as a composite score for each health facility by combining the scores of all indicators. Moreover, a mean percentage score was calculated (composite score*100/total number of indicators in checklist) for each facility and presented with Standard Deviation (SD).

- Readiness of health facilities to provide DFHC (Table 3)

The readiness of a health facility to provide services for people with disabilities was assessed across four domains (i.e. information, communication, infrastructure and capacity of healthcare providers). Data collected from structured interviews with health facility managers were analyzed, which had 30 indicator variables under each domain. Presence of an indicator in a facility was recorded as ‘Yes/No’ and for each response a score was assigned where ‘Yes = 1 & No = 0″. The number of indicators present under a domain formed the Domain specific score (sum of indicators for each domain/total number of indicators of that domain) at each health facility. Furthermore, domain specific mean percentage scores were calculated for all DFHC indicators at each type of health facility, using the following formula: number of individual indicator(s) present in each health facility *100/total number of each domain indicator(s). Then the average mean percentage score was calculated for all four domains of each health facility type.

- Satisfaction level of persons with disability (Table 2)

**Table 2:** Satisfaction level on information, communication and accessibility to health facilities by persons with disabilities

***Satisfaction level of the persons with disabilities***						
		**Very satisfied**	**Satisfied**	**Neutral**	**Very dissatisfied**	**Dissatisfied**
		n (%)	n (%)	n (%)	n (%)	n (%)
1	**Getting Information (*n* = 307)**	1 (0.3)	46 (15.0)	75 (24.4)	174 (56.7)	11(3.6)
2	**Communicating with HCPs (*n* = 307)**	-	52 (16.9)	77 (25.1)	165 (53.7)	13 (4.2)
***Accessibility status of the health facility infrastructure by the persons with disabilities***						
	**Infrastructural points of the health facilities**	Very accessible	Accessible	Neutral	Very inaccessible	Inaccessible
1	Entrance (*n *= 307^a^)	-	40 (13.0)	85 (27.7)	156 (50.8)	26 (8.5)
2	Ticket counter (*n* = 222^a^)	-	21 (9.5)	42 (18.9)	130 (58.6)	29 (13.1)
3	Reception area (*n* = 151^a^)	-	28 (18.5)	35 (23.2)	81 (53.6)	7 (4.6)
4	Out-patient department (For applicable cases) (*n* = 231^a^)	-	50 (21.6)	56 (24.2)	117 (50.6)	8 (3.5)
5	Diagnostic room (For Applicable Cases) (*n* = 101^a^)	-	22 (21.8)	29 (28.7)	49 (48.5)	1 (1.0)
6	Hospital Ward (For applicable cases) (*n* = 72^a^)	-	4 (5.6)	24 (33.3)	42 (58.3)	2 (2.8)
7	Pharmacy (*n* = 117^a^)	4 (2.1)	65 (34.2)	36 (18.9)	82 (43.2)	3 (1.6)
8	Toilet (*n* = 142^a^)	-	2 (1.4)	15 (10.6)	79 (55.6)	46 (32.4)

^a^Those who used the specific infrastructural points of the health facilities

To assess the level of satisfaction of persons with disabilities with access to information, communication, and eight selected infrastructure points, a five-point Likert scale with values ranging from 1 (strongly disagree) to 5 (strongly agree) was used. The level of satisfaction for each category is presented as frequency and percentage.

Qualitative data were analyzed using a thematic approach. The audio-recording and hand-written notes were transcribed and then the transcripts were checked and validated. Finally, the data were charted and interpreted using a predetermined thematic framework. The framework included opinion on current healthcare delivery system, perception on DFHC, barriers to providing DFHC, and recommendation to improve disability friendliness of health facilities as major themes.

## Results

The results of the study are given in three sections: the current situation, barriers, and suggestions for the health facilities in terms of DFHC. In each section, notable findings of accessible information and communication, accessible infrastructure and capacity of HCPs are described. Satisfaction level and accessibility status of accessing services from facilities including perception of healthcare providers on current status of disability friendliness in the health facilities are given in a separate section.

### Current situation of the health facilities in terms of DFHC

#### Access to information

In the structured interviews, only one fifth of the persons with disabilities reported that they obtained information through their own efforts. The majority of respondents (85.7%) stated that in most cases their caregivers collected information on their behalf. Almost all (96.6%) of the health facility managers mentioned that caregivers collected information on behalf of the persons with disabilities, while only few (8.8%) mentioned about collecting information directly from health facility staff. However, none of the persons with disabilities informed that they used any assistive device or signage to obtain necessary information.

#### Access to communication

Similar to access to information, the healthcare providers could not communicate with the persons with disabilities without their caregivers. As a means of communication, half of the persons with disabilities (51.1%) claimed that the communication was made orally; and among them (87.0%) reported that they communicated through their caregivers (data not shown). In one of the FGDs, a child (below 15 years of age) mentioned that during the consultation session, he shared his problems with his parents and then his parents communicated with the doctor. One heath care provider in IDI stated *“I always wait for a relative to come with the people with disabilities as I do not understand their sign or body language. Sometimes persons with disabilities use some language which their family members can understand.”*

#### Accessibility of infrastructure

Besides access to information and communication, to observe access to infrastructure the study also assessed 150 health facilities against13 different points regarding infrastructure.

Total mean percentage score of all infrastructure areas considering all healthcare facilities was (20 ± 13.7). The tertiary level healthcare facilities scored the highest (46.0 ± 10.3) and the primary healthcare facilities obtained the lowest score (17.1 ± 11.6). When the infrastructure points of all types of health facilities were analysed together, the mean percentage score for the majority of these points was below 20. The consultation rooms of healthcare providers received the highest mean percentage score (44.0 ± 34.2) followed by entrance (38.7 ± 25.3), general areas (33.8 ± 24.6) and signage (27.3 ± 23.3), while the elevators scored the lowest mean percentage (5.6 ± 5.0). The score was high (63.9 ± 39.7) in tertiary healthcare facilities. It is worth mentioning that elevators were not available in primary healthcare settings (Table 1).

**Fig. 3:**
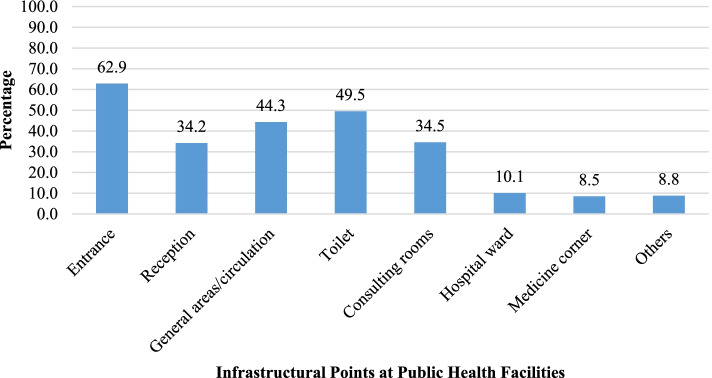
Distribution of infrastructural points where people with disabilities faced difficulties

During structured interviews, almost two-thirds (62.9%) of the people with disabilities reported that during visits to a health facility they faced difficulties at the entrance. About half of the respondents (49.5%) had difficulties during use of a toilet. Other areas where the persons with disabilities had problems include general areas/circulation (44.3%), consulting rooms (34.5%) and reception areas (34.2%) (Fig. 3). The barriers for using toilets as reported by the people with disabilities in FGDs included inadequate space around doors for wheelchairs, unusable door knobs, inappropriate sink heights, faucet handles, the absence of a high commode, and inadequate space for using a wheelchair inside the toilet.

#### Capacity of the healthcare providers

The health facility managers were asked about the capacity of the HCPs to provide disability friendly healthcare during the structure interviews. Among the four selected specialized hospitals only two had trained designated staff to provide information to the persons with disabilities and could communicate with them.

### Barriers of the health facilities to provide DFHC

#### Access to information

Through IDIs and FGDs with persons with disabilities and their caregivers it was identified that a lack of training among HCPs was the main reason for information inaccessibility. Moreover, the HCPs in IDIs reported that they were not aware of anything about “accessible information” for people with disabilities.

One of the HCPs stated that *“We provide information in the ticket counter, outpatient department or residential medical officer`s room but we do not have any specific information centre for the people with disabilities*.”

#### Access to communication

Lack of trained facility staff was identified as one of the major reasons for poor communication, as stated by both service providers and service recipients during different qualitative interviews. The NGO workers stated that neither the service providers nor the service recipients or their caregivers were educated on sign language. In FGDs, the persons with disabilities mentioned that the health service providers have a lack of knowledge on disability care. A woman with disability stated – *“One day I saw a person who cannot speak properly was trying to say something to a doctor but he did not understand what the person was saying. The doctor then said he is busy and referred him to another doctor.”*

#### Accessible infrastructure

In KIIs, most of the policy makers were concerned about the current infrastructure of the health facilities in Bangladesh. One of the KII respondents mentioned that there was a plan to provide service points in the health facilities for the persons with disabilities but were yet to implement Another participant of KIIs mentioned – *“There is not enough space to provide seating arrangements for the persons with disabilities at the facilities.”* In FGDs, women with disabilities shared that the general population do not receive health services properly in the hospital so for people with disability it is out of the question.

### Perception of persons with disabilities on DFHC

A total of 185 (60.3%) patients claimed that they were dissatisfied with the services of getting necessary information from the facilities. Similarly, 57.9% of the people with disabilities were dissatisfied in communicating with the HCPs (Table 2).

Among the persons with disabilities, the majority (ranging from 44.8 to 88.0%) felt that almost all the infrastructural points of the health facilities were inaccessible to them. Toilets were mentioned as the most inaccessible (88.0%) infrastructure point, followed by the ticket counter (71.7%).

### Perceptions of the healthcare providers on DFHC

According to the response from the health facility managers the highest score was observed against infrastructure which was (29.3 ± 20.5) and the lowest (0.93 ± 7.1) mean percentage was capacity of the HCPs to provide DFHC among the four domains. Overall, the score was found low in all domains of DFHC in all tiers of health facilities (Table 3).

**Table 3:** Mean percentage score of four domains of DFHC in different levels of facilities as reported by the healthcare providers

Domains of DFHC	All Facilities (*n* = 150)	Tertiary level healthcare (*n* = 9)	Secondary level healthcare (*n* = 17)	Primary level healthcare (*n* = 124)
Information	14.6 ± 6.22	18.0 ± 11.0	12.5 ± 4.4	14.6 ± 5.9
Communication	18.2 ± 4.8	22.2 ± 8.3	17.6 ± 4.0	18.0 ± 4.5
Infrastructure	29.3 ± 20.5	38.4 ± 21.6	32.0 ± 30.3	28.3 ± 18.7
Capacity of healthcare providers	0.93 ± 7.1	6.7 ± 20.0	3.5 ± 14.5	0.16 ± 1.7
All domains	15.7 ± 5.9	21.3 ± 6.6	16.4 ± 9.4	15.2 ± 5.1

[Results are expressed as Mean percentage and 1 Standard Deviation]

### Recommendations from the respondents to make the health facilities disability friendly

The study also gathered suggestions from the health policy makers, healthcare providers, disability related NGO workers, community leaders, and people with disabilities including women, children and their caregivers to identify elements for making facilities disability friendly through KIIs, IDIs and FGDs. Besides structured interviews were carried out among health administrators and persons with disabilities to obtain their inputs on DFHC.

#### Accessible information

Almost all (98.4%) of the persons with disabilities suggested to provide training to the health facility staff for improving their skills of providing information to their patients including persons with disabilities. Slightly over 40.0% of the respondents suggested using signage and pictorials to provide information. One of the caregiver respondents in the FGDs suggested the following -

“Clear signage is very important for every one including people with disabilities.”

Moreover, the HCPs in IDIs recommended that every health facility should have a welcome desk and there should be a designated person at all times to provide information to the patients or their caregivers including persons with disabilities.

#### Accessible communication

**Fig. 4:**
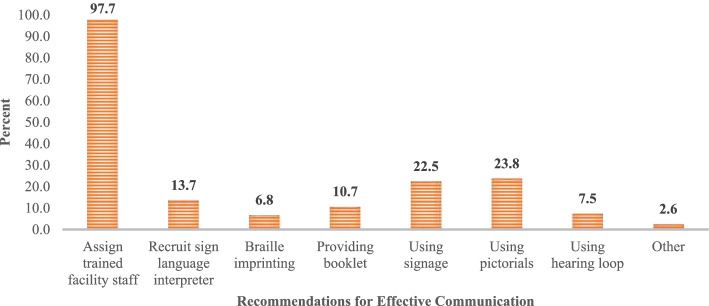
Response of the people with disabilities on the suggestions for mode of communication with healthcare providers. * Multiple responses were allowed during their interviews

Similar to accessible information, almost all (98.0%) people with disabilities in their structured interviews proposed to assign trained staff for effective communication between healthcare providers and service recipients. Other suggestions they made were using signage (22.5%) and pictorials (23.8%) and recruiting sign language interpreters (13.7%) (Fig. 4).

Moreover, most of the healthcare providers in IDIs suggested developing human resources and the provision of communicative devices for people with disabilities in the health facilities.

#### Access to infrastructure

Policy makers in KIIs provided diverse suggestions to improve the current situation of infrastructure at health facilities. One of the key informants narrated as follows:

“A little change in the health facility infrastructure may make the facility accessible for the people with disabilities. Like toilet, ramp, entrance and others.” –

Community leaders, persons with disabilities and their caregivers in the IDIs suggested ensuring the availability of handrails, wheel chairs and trollies in the facilities. They also mentioned that there should be provision of ramps in each health facility.

#### Capacity of the healthcare providers

Almost all HCPs of all types of health facilities suggested raising awareness of the facility staff on disability friendly healthcare. Almost all (80.0 to 100.0%) HCPs of specialized hospitals down to MCWCs, and 50.0 to 60.0% from UHCs and below suggested tailored training including braille, sign language, and communication skill for different types of disabilities.

## Discussion

This study identified that persons with disabilities have poor access to health facilities to obtain information and effective communication. The infrastructure of the majority of the facilities were not accessible to this population. Moreover, the healthcare providers were not capable of providing disability friendly healthcare.

This research is the first of its kind to identify barriers for persons with disabilities to obtaining disability friendly healthcare in terms of access to information and communication, access to infrastructure and capacity of the health providers in Bangladesh. The study deployed a mixed method approach, which helped to determine the accessibility issues in quantitative form, and the qualitative techniques thoroughly explored service providers and recipients’ perceptions, barriers to accessible healthcare, and suggestions to improve the current situation.

This study showed that the majority of the persons with disabilities had difficulties in obtaining information and communicating with service providers. In both cases, people with disabilities had to rely on their caregivers.

Studies conducted in low- and middle-income countries of Asia [12] and Sub-Saharan Africa [13], have found that the system of providing information was not very organized, and the format was also inaccessible to persons with disabilities. When audio was used for providing information to persons with disabilities no visuals were included, similarly the posters were without audio content. In other words, during development of information and communication materials alternative formats were not considered for different types of disabilities. To provide proper treatment for people with disabilities, health facilities must provide information in accessible formats, assisting communication with the healthcare providers to improve. These domains, accessible information and communication, are also described in the UNCRPD and World Health Organization (WHO) global disability action plan 2014–2021 with an emphasis on utilization of mobility aids and assistive technologies [5, 14]. To keep in line with these two international commitments, many developed countries (Ireland, Malaysia and Sri Lanka) utilized several key measures including using pictorials, booklets, sign language and braille to provide accessible information and communication [6, 7]. Although, the accessibility of information and communication are included in the disability action plan of Pakistan and India, such plans are not yet in place [15]. Similarly, in Bangladesh, in different national policies, plans and programmes the rights of disabled people regarding receiving information and effective communication have also been considered. Utilizing audio and braille in hospitals and, where necessary, engaging a sign language interpreter, are mentioned in the ‘Persons with Disabilities Rights and Protection Acts 2013’, however, none of these practices were observed in this study [16].

This study also observed the current context of health facilities at an infrastructure level, which is mostly considered when the concern is healthcare for persons with disabilities. In this study at every level of the healthcare service, i.e., from primary to tertiary, most of the infrastructure points of the health facilities in terms of its accessibility scored very low (the mean percentage score was below 20). Although, there is no standard cut-off value to identify the level of accessibility, this low score indicates the poor accessibility to the infrastructure points by the persons with disabilities in Bangladesh.

In a limited number of secondary and tertiary level health facilities, certain infrastructure service points to some extents were found accessible to persons with disabilities in the study, whereas in the primary healthcare settings the situation was worse. Similar findings were also observed in other studies in Bangladesh and other countries. Research conducted in Bangladesh in 2015 reported that infrastructure of health facilities for people with disabilities was found quite inaccessible, especially for rural settings. The report also showed that none of the health facilities had any disability friendly washrooms [17]. In rural areas of South Africa, persons with disabilities faced similar difficulties in accessing healthcare due to structural barriers of the health facilities [18].

In the United States of America (USA), persons with mobility disabilities found medical facilities and services more inaccessible than other types of disabilities [19]. For ensuring full and equal access to healthcare services and facilities, the Americans with Disabilities Act (ADA) emphasized and adopted 2010 ADA Standards for Accessible Design to make medical infrastructure accessible to persons with disabilities [20]. In these accessibility standards a set of minimum requirements for newly constructed facilities were established for example, accessible parking spaces, curb ramps at entrance, wide doorways, restrooms with enough space for wheelchairs, grab rails inside toilets, tactile floorings, signage with braille and accessible medical equipment [21].

In other countries it has been emphasized that during construction of a health facility, disability friendly aspects should be considered right from the design phase. For example, in Wellawaya, Sri Lanka there were some initiatives taken to make accessible health services such as proper ramps, adequate sign language, specific seating arrangements and queue for persons with disabilities [22]. The guidelines for accessible healthcare in Ireland and Malaysian Uniform Building By-Laws (UBBL) and other standard regulations enforce making health facilities disability friendly. The guidelines and regulations provided specific measurements and design for ramps (1:20 slope ratio), elevators including braille button and handrails, stairs with standard height and width, entrances with stair free pathways, accessible parking, signage with pictures and toilets for comfortable utilization by a person with disabilities [6, 7].

The study also found that there was no designated staff to provide information and communicate with persons with disabilities, and the healthcare providers were not trained to do so. In this study, the health policy planners and managers, service providers, NGO workers and the service recipients proposed to train facility staff on accessible information and communication for persons with disabilities. The ADA, Civil Rights Acts of USA also suggested to provide training to the healthcare professionals for accessible healthcare for the persons with disabilities [20]. Similarly, a toolkit on disability for Africa gave importance on training of doctors, nurses and health professionals to provide inclusive healthcare for persons with disabilities [8]. In Bangladesh, training to the HCPs also get prioritized for providing accessible healthcare and treatment to persons with disabilities in the ‘Persons with Disabilities Rights and Protection Acts 2013’. However, this training programs did not holistically include DFHC.

The study also assessed the satisfaction level of and accessibility status of selective infrastructure points of health facilities by the persons with disabilities in terms of DFHC. The majority of persons with disabilities were dissatisfied with the access to information and communication. In addition, all of the selected (eight) infrastructure points, those that are the most imperative for a health facility, were found inaccessible by persons with disabilities. A study conducted in the Kurigram district of Bangladesh reported similar dissatisfaction by persons with disabilities for obtaining information and the manner of communication by the HCPs toward people with disabilities [23]. Stereotypical attitudes of healthcare staff were identified as one of the main reasons for dissatisfaction associated with getting proper information by the patient with disabilities in Pakistan. Faulty design of health facility infrastructure was another reason for dissatisfaction as expressed by persons with disabilities [24]. In this study, healthcare providers opined that the health facilities were not ready to serve the persons with disabilities in relation to the four domains of DFHC.

The findings of this study provided opportunities for improvement of the existing situation of health facilities to provide DFHC. In Bangladesh, though there are policies, strategies and Acts in which DFHC are recommended to some extent, there has been no costed action plan or implementation. DFHC should be included in the national health policy, accordingly programs and budgets need to be included in the operation plan for DFHC. To keep in line with policy, a national guideline on DFHC for the HCPs should be developed for capacity building. Each health facility should develop DFHC guiding principles following a standard guideline which should be stated in the national policy. The policy should be displayed publicly at the relevant areas. Suitable signage and assistive device should be in place and health facility staff should be trained on accessible information and effective communication. Renovation or modification of the existing infrastructure service points, where possible, should be done to improve accessibility of persons with disabilities. All infrastructure service points should be accessible and equipped with all types of supportive or protective devices. A standard architectural and structural design of disability friendly health facilities should be developed for constructing future health facilities. A structured monitoring and evaluation system should be in place to assess the progress of DFHC in Bangladesh by the competent authority.

### Limitations and strengths of the study

The study has few limitations. A major drawback of the study is that it doesn’t provide information on the challenges of service recipients according to the nature and severity of their disability. The study is also unable to compare the dissatisfaction level of persons with disability to general population in Bangladesh. Furthermore, it was unable to document the sociodemographic characteristics of the people with disabilities surveyed, limiting clarity on population characteristics and generalizability. Additionally, the study could not compare the situation in urban and rural healthcare settings for persons with disabilities in detail as the sample size for the urban health facilities was not large enough.

Despite these limitations, the study offers recent, novel, and significant evidence on the current situation of Bangladesh’s public healthcare facilities in terms of disability friendliness. It involved all levels of healthcare facilities, and investigated both service provision at facilities and consumer experience, providing a comprehensive scenario. Thus, it serves as an important evidence base for developing interventions to promote disability friendly health services in Bangladesh and other similar contexts.

## Conclusion

The study revealed that most of the public health facilities in Bangladesh, from specialised hospitals to community clinics, were not disability friendly in terms of access to information and communication, access to infrastructure and capacity of the healthcare providers. However, there is scope to improve the current situation. A national disability friendly policy including implementation guidelines for health facilities should be developed to improve access to healthcare by persons with disabilities.

## Supplementary Information

**Additional file 1.** Questionnaires for the persons with disabilities.

## Data Availability

The datasets generated and/or analyzed during the current study are not publicly available since another manuscript and a policy review are being prepared from the findings of this dataset. However, datasets are available from the corresponding author on reasonable request.
