# Supplementary material for: Disability-friendly healthcare at public health facilities in Bangladesh: a mixed-method study to explore the existing situation
Source: BMC Health Serv Res. 2022 Sep 20;22:1178. doi: 10.1186/s12913-022-08538-6 (PMC9490997; doi:10.1186/s12913-022-08538-6)
Supplement: Supplementary file 1 — Additional file 1. Questionnaires for the persons with disabilities. [file 12913_2022_8538_MOESM1_ESM.pdf]

2018 Questionnaires for the Persons with Disabilities (PWDs)

Form-10

Mainstreaming Disability Friendly Health Services (DFHS) focusing MNC&AH program
Centre for injury prevention and research, Bangladesh (CIPRB)

Form 10- Structure questionnaire-Persons with disabilities (PWDs)

Name of research assistant-

Date of data collection-

**dd**	**mm**	**yy**

Type of survey location	Name	Code	Time of interview
District		☐☐	**Start time of interview-** ☐☐ : ☐☐
Upazila		☐	
Union		☐	
Health facility (please tick the health facility and write the code)	Specialized hospital	☐☐	**End time of interview-** ☐☐ : ☐☐
	Medical College hospital		
	District hospital		
	Maternal & child welfare centre		
	Upazila health complex		
	Union health & family welfare centre / Union health centre/ Union sub-centre		
	Community clinic		
Person with disability		☐	
Unique code	☐☐☐☐☐☐☐		
Types of disability (Multiple answer allowed)	01- Autism/ Autism Spectrum Disorder<br>02- Physical<br>03- Psychosocial /Mental<br>04- Visual<br>05- Speech<br>06- Intellectual<br>07- Hearing<br>08- Deaf Blind<br>09- Cerebral Palsy<br>10- Down Syndrome<br>11- Others, Specify	☐☐<br>☐☐<br>☐☐	

2018 Questionnaires for the Persons with Disabilities (PWDs)

Form-10

Questionnaires		Code
**1. Perception/Attitude on Disability Friendly Health Services (DFHS)**		
1.1	Do you think that all people with disabilities have the right to get equity health services in a facility?<br>1-Yes<br>2-No	
1.2	Do you think it is ok to not getting equal health services in a facility?<br>1-Yes<br>2-No	
1.3<br>If yes go to 1.4, if no go to 1.5	Do you/ your patient with disabilities use health services regularly from any health facility?<br>1-Yes<br>2-No	
1.4	If yes, from where/which facility have you/ your patient with disabilities received the health services?(Try to understand the mostly seeking tier of health facilities from primary to tertiary level)<br>(Multiple answer allowed)<br>1-Mentioned<br>2-Not mentioned	
	a. Specialized Hospitals	
	b. Medical College Hospitals	
	c. District Hospitals	
	d. Maternal and Child Welfare Centres	
	e. Upazila Health Complexes	
	f. UHFWC/Union Sub-centre	
	g. Community Clinic	
	h. Other-specify____	
1.5	If no, why are not you/your patient with the disabilities using health services from health facility?<br>(Multiple answer allowed)<br>1- Mentioned<br>2- Not mentioned	
	a. Do not know about the right to get health services	
	b. Negative attitude of the health service providers	
	c. No transport facility	
	d. No available health facility around the village	
	e. No assistance to help to come health facility	
	f. Not affordable to get health services	
	g. Others, specify<br>____	

1.6	When was the last time you/ your patient with disabilities received health services from a health facility?(Response in months)<br><br>____	
1.7	What type of health services did you/ your patient with disabilities sought in your last visit / currently seeking?<br>(Multiple answer allowed)<br>1-Mentioned<br>2-Not mentioned	
	a. General service	
	b. Screening	
	c. Maternal service	
	d. Neonatal service	
	e. Child service	
	f. Immunization	
	g. Adolescent service	
	h. Other, please specify<br>____	
1.8<br>**If not yes go to 2.1**	Have you/ your patient with disabilities heard of any Policy/Act/Law/Rights related to disability regarding health services?<br>1-Yes<br>2-No<br>3-Donot know<br>4-Do not understand	
1.9	If yes, mention the title?(Multiple answer allowed)<br>1-Mentioned<br>2-Not mentioned	
	a. National Health Policy 2011	
	b. Disability Welfare Act 2001	
	c. Disabilities Rights and Protection Acts 2013	
	d. Other, please specify<br>____	
1.10	What are you/ your patient with disabilities perception about the Policies/Acts/Laws/Rights? (Multiple answer allowed)<br>1-Mentioned<br>2-Not mentioned	
	a. Equity to get health services	
	b. Free health service	
	c. Accessibility to get health services	
	d. Not applicable	
	e. Other-specify____	

2018 Questionnaires for the Persons with Disabilities (PWDs)

Form-10

2. Situation Assessment on DFHS		
	**Accessible Information**	
2.1	How did you/your patient with disabilities get any information about the services that you needed in the facilities? (Multiple answer allowed)<br>1-Mentioned<br>2-Not mentioned	
	a. By the help of health service provider	
	b. Himself / Herself	
	c. By the help of caregiver	
	d. Other, please specify<br>____	
2.2<br>**If yes go to 2.3**	Did you/your patient with disabilities get any assistive device for information about the health services?<br>(Multiple answer allowed)<br>1-Yes<br>2-No	
2.3	If yes, mention the device?(multiple answer allowed)<br>1-Mentioned<br>2-Not mentioned	
	a. Booklet	
	b. Braille inscription	
	c. Pictorial graph	
	d. Hearing aid	
	e. External sign	
	f. Internal sign	
	g. Other, please specify<br>____	
2.4	How satisfied are you to have all the information need to get health services?<br><br>5-Very satisfied 4-Satisfied 3-Neutral 2-Dissatisfied 1-Very dissatisfied	
	**Accessible Communication**	
2.5	How did the health service providers communicate with you/ your patient with disabilities? (Multiple answer allowed)<br>1-Mentioned<br>2-Not mentioned	
	a. Facility staff use sign language to communicate	
	b. Facility staff communicate through braille	
	c. Oral communication	
	d. Facility have hearing loop	
	e. Through caregiver	
	f. Other, please specify<br>____	

2.6	How satisfied are you to communicate with health service providers need to get health services?<br><br>5-Very satisfied 4-Satisfied 3-Neutral 2-Dissatisfied 1-Very dissatisfied	
2.7<br>**If not yes go to 2.9**	Did you have any separate noise free room to communicate with you/your patient with disabilities in any facility?<br>1- Yes<br>2- No<br>9- Cannot remember	
2.8	If yes, which facility? (Multiple answer allowed)<br>1- Mentioned<br>2- Not mentioned	
	a. Specialized Hospitals	
	b. Medical College Hospitals	
	c. District Hospitals	
	d. Maternal and Child Welfare Centres	
	e. Upazila Health Complexes	
	f. UHFWC/Union Sub-centre	
	g. Community Clinic	
	h. Other, specify<br>____	
	**Accessible Infrastructure**	
2.9	In which part have you/ your patient with disabilities faced access challenges in the infrastructure of a facility?<br>(Multiple answer allowed)<br>1-Mentioned<br>2-Not mentioned	
	a. Entrance	
	b. Reception	
	c. General areas and circulation	
	d. Toilet facilities	
	e. Consulting and treatment rooms	
	f. Hospital wards	
	g. Signs and notices	
	h. Elevator	
	i. Safe evacuation	
	j. Medicine corner	
	k. Other, specify<br>____________________________	

2018 Questionnaires for the Persons with Disabilities (PWDs)

Form-10

	**Entrance**	
2.10	Did you/ your patient with disabilities have any accessible wheelchairs at the entrance?<br>1-Yes<br>2-No<br>9-N/A	
2.11	Did you/ your patient with disabilities find entry and exit point that is clearly sign-posted close to the entrance to the building?<br>1-Yes<br>2-No<br>9-N/A	
2.12	Did you/ your patient with disabilities face any obstructions at the entrance?<br>1-Yes<br>2-No<br>9-N/A	
2.13	Are there door/lock pull handles at the entry/exit point?<br>1-Yes<br>2-No<br>9-N/A	
2.14	How accessible the entrance of the facility for you?<br><br>5-Very accessible 4- accessible 3-Neutral 2- inaccessible 1-Very inaccessible	
	**Collecting Ticket**	
2.15<br>**If not yes go to 2.20**	Did you/ your patient with disabilities collect ticket from any counter?<br>Yes-1<br>No-2<br>N/A-9	
2.16	If yes, what type of counter you have found?<br>1- Mentioned<br>2- Not mentioned	
	1- Common counter	
	2- Separate counter for PWDs	
	3-Two-tier counter	
	4- No counter	
	5- Other, specify<br>____	
2.17	Did you face any difficulties to collect ticket from the counter?<br>1-Yes<br>2-No	
2.18	If yes, then what type of problem have you faced?<br>(Multiple answer allowed)<br>Mentioned-1<br>Not mentioned-2	
	a. Finding the counter	
	b. No ramp or step free pathway	
	c. Long queue	

	d. Absence of ticket giver	
	e. Pushing/Shouldering	
	f. Understanding the content of the ticket	
	g. Other-specify ______________________	
2.19	How accessible the counter for collecting the ticket for you?<br><br>5-Very accessible 4- accessible 3-Neutral 2- inaccessible 1-Very inaccessible	
	**Reception**	
**2.20 If no go to 2.29**	Did you find any reception desk in a facility?<br>1-Yes<br>2-No	
2.21	If yes**,** How did you find the reception/information desk in a facility?<br>(Multiple answer allowed)<br>1-Mentioned<br>2-Not mentioned	
	a. Braille inscription	
	b. Tactile floor direction	
	c. Sign direction	
	d. Caregiver support	
	e. Hospital staff assistance	
	f. colour coded direction	
	g. Other-Specify ______________________	
2.22	How suitable were the reception desks to use?<br>(Multiple answer allowed)<br>1-Mentioned<br>2-Not mentioned	
	a. height of the reception desk allowing people to stand	
	b. height of the reception desk allowing people to sit	
	c. height of the reception desk allowing use a wheelchair while meeting at reception.	
	d. Other-specify ______________________	
2.23	Did you face any difficulties to communicate with the receptionist?<br>1-Yes<br>2-No<br>9-N/A	
2.24	If yes, what difficulties have you faced?(multiple answer allowed)<br>1- Mentioned<br>2-Not mentioned	
	a. receptionist did not cooperate properly	

	b. did not understand what he/she said	
	c. no one was there to communicate	
	d. Other-specify ________________________________	
2.25	Did the receptionist use any communicative/assistive device/assistance?<br>1-Yes<br>2-No<br>9-N/A	
2.26	If yes, can you please mention the device/devices?<br>(multiple answer allowed)<br>1-mentioned<br>2-not mentioned	
	a. Braille	
	b. Sign language	
	c. Hearing loop	
	d. Audio	
	e. Pictorial	
	f. Caregiver support	
	g. Others-specify ________________________________	
2.27	Have you found the reception area accessible?<br><br>5-Very accessible 4- accessible 3-Neutral 2- inaccessible 1-Very inaccessible	
2.28	Did you have the effective communication with the receptionist?<br><br>5-Very effective 4- effective 3-Neutral 2- ineffective 1-Very ineffective	
	**Out-patient department (For applicable cases)**	
**2.29 If not yes go to 2.40**	Did you have to go to out-patient department to get services?<br>1-Yes<br>2-No<br>9-N/A	
2.30	If yes, How did you find the out-patient department?<br>(Multiple answer allowed)<br>1-Mentioned<br>2-Not mentioned	
	a. By yourself	
	b. By your caregiver	
	c. By hospital staff	
	d. Sign/symbol	
	e. Braille inscription	
	f. Tactile floor direction	
	g. Handrail	
	h. Not applicable	
	i. Colour coded direction	

	j. Other-specify ____	
2.31 **If no go to 2.33**	Whether you/ your patient were referred to another facility or institution for further treatment/assistance?<br>1-Yes<br>2-No<br>9-N/A	
2.32	If yes, Was transport /ambulance provided?<br>1-Yes<br>2-No<br>9-N/A	
2.33	Was there enough space to remain on your wheel chair in the room (for wheelchair user)?<br>1-Yes<br>2-No<br>9-N/A	
2.34 **If no go to 2.36**	Did the doctor help you to understand the prescription accurately?<br>1-Yes<br>2-No<br>9-N/A	
2.35	If yes, How? (Multiple answer allowed)<br>1- mentioned<br>2-not mentioned	
	a. using assistive device	
	b. Sign language interpreter	
	c. By her/himself	
	d. By caregiver	
	e. By hospital staff	
	f. Others-specify ____	
2.36 **If no go to 2.38**	Did the doctor use any assistance/device to communicate?<br>1-Yes<br>2-No<br>9-N/A	
2.37	If yes, mention the device?<br>1- Mentioned<br>2-Not mentioned	
	a. Braille	
	b. Sign language	
	c. Hearing loop	
	d. Audio	
	e. Pictorial	
	f. Others-specify ____	
2.38	Have you found the outpatient department area accessible?<br><br>5-Very accessible 4- accessible 3-Neutral 2- inaccessible 1-Very inaccessible	
2.39	Did you have the effective communication with the health service providers?	

	5-Very effective 4- effective 3-Neutral 2- ineffective 1-Very ineffective	
	**Diagnostic room (For Applicable Cases)**	
2.40<br>**If not yes go to 2.49**	Did you have to go to a diagnostic room to get services?<br>1-Yes<br>2-No<br>9-N/A	
2.41	If yes, How did you find the diagnostic room?<br>(Multiple answer allowed)<br>1-Mentioned<br>2-Not mentioned	
	a. By yourself	
	b. By your caregiver	
	c. By hospital staff	
	d. Sign/symbol	
	e. Braille inscription	
	f. Tactile floor direction	
	g. Handrail	
	h. Not applicable	
	i. Other-specify<br>____	
2.42	Was there enough space to remain on your wheel chair in the room (for wheelchair user)?<br>1-Yes<br>2-No<br>9-N/A	
2.43<br>**If no go to 2.45**	Did the health service providers help you to understand the instruction for test accurately?<br>1-Yes<br>2-No<br>9-N/A	
2.44	If yes, how did the health service providers instructed you?<br>(Multiple answer allowed)<br>1- mentioned<br>2- not mentioned	
	a. By caregiver	
	b. By him/herself	
	c. By using assistive device	
	d. By hospital staff	
	e. Other, specify<br>____	
2.45<br>**If no go to 2.47**	Did they use any assistance/device to communicate?<br>1-Yes<br>2-No<br>9-N/A	
2.46	If yes, mention the device? (multiple answer allowed)<br>1-mentioned<br>2-not mentioned	

	a. Braille	
	b. Sign language	
	c. Hearing loop	
	d. Audio	
	e. Pictorial	
	f. Others-specify ______________________________	
2.47	Have you found the diagnostic room accessible?<br><br>5-Very accessible 4- accessible 3-Neutral 2- inaccessible 1-Very inaccessible	
2.48	Did you have the effective communication with the health service providers in there?<br><br>5-Very effective 4- effective 3-Neutral 2- ineffective 1-Very ineffective	
	**Hospital Ward (For applicable cases)**	
2.49<br>**If not yes go to 2.56**	Did you have to go to hospital ward to get services?<br>1-Yes<br>2-No<br>9-N/A	
2.50	If yes, How did you find the hospital ward?<br>(Multiple answer allowed)<br>1-Mentioned<br>2-Not mentioned	
	a. By yourself	
	b. By your caregiver	
	c. By trained hospital staff to communicate with PWDs	
	d. Sign/symbol	
	e. Braille inscription	
	f. Tactile floor direction	
	g. Handrail	
	h. Not applicable	
	i. Other-specify ______________________________	
2.51	Were there adjustable beds in the room?<br>1-Yes<br>2-No<br>9-N/A	
2.52	Did they suggest any follow up plan during treatment/discharge?<br>1-Yes<br>2-No<br>9-N/A	

2.53<br>**If no go to 2.55**	Did the health service provider clearly mention about the follow up plan?<br>1-Yes<br>2-No<br>9-N/A	
2.54	If yes, how they communicate with you? (multiple answer allowed)<br>1-mentioned<br>2-not mentioned	
	a. By caregiver	
	b. By trained hospital staff	
	c. By him/herself	
	d. By using assistive device	
	e. Other, specify<br>____	
2.55	Have you found the hospital ward accessible?<br><br>5-Very accessible 4- accessible 3-Neutral 2- inaccessible 1-Very inaccessible	
2.56	Did you have the effective communication with the health service providers in there?<br><br>5-Very effective 4- effective 3-Neutral 2- ineffective 1-Very ineffective	
	**Pharmacy**	
2.57<br>**If not yes go to 2.63**	Did you have to go to pharmacy to get medicines?<br>1-Yes<br>2-No<br>9-N/A	
2.58	If yes, How did you find the pharmacy?<br>(Multiple answer allowed)<br>1-Mentioned<br>2-Not mentioned	
	a. By yourself	
	b. By your caregiver	
	c. By hospital staff	
	d. Sign/symbol	
	e. Braille inscription	
	f. Tactile floor direction	
	g. Handrail	
	h. Not applicable	
	i. Other-specify<br>____	
2.59	Did pharmacist help you to understand the dosage of medicine?<br>1-Yes<br>2-No<br>9-N/A	
2.60	How did the pharmacists explain to you the instruction?<br>(Multiple answer allowed)<br>1-Mentioned<br>2-Not mentioned	

	a. Orally	
	b. Sign language	
	c. Braille	
	d. Care-giver	
	e. Other-specify ______________________________	
2.61	Have you found the pharmacy area accessible?<br><br>5-Very accessible 4- accessible 3-Neutral 2- inaccessible 1-Very inaccessible	
2.62	Did you have the effective communication with the pharmacist in there?<br><br>5-Very effective 4- effective 3-Neutral 2- ineffective 1-Very ineffective	
	**Elevator(applicable cases)**	
2.63<br>**If not yes go to 2.67**	Have you ever used the elevator in a facility?<br>1-Yes<br>2-No<br>9-N/A	
2.64	Did you face any difficulties to enter into elevator?<br>1-Yes<br>2-No<br>9-N/A	
2.65	If yes, then what type of problem you have faced?<br>(Multiple answer allowed)<br>1-Mentioned<br>2-Not mentioned	
	a. Entrance space was not sufficient	
	b. Floor area space was not sufficient	
	c. Elevator button was out of reach	
	d. No braille code in the button	
	e. No voice projection	
	f. Other- Specify ______________________________	
2.66	Have you found the elevator accessible?<br><br>5-Very accessible 4- accessible 3-Neutral 2- inaccessible 1-Very inaccessible	
	**Toilet**	
2.67<br>**If not yes go to 2.72**	Did you use any toilet in a facility?<br>1-Yes<br>2-No<br>9-N/A	
2.68	If yes, Did you find any accessible toilet for PWDs?<br>1-Yes<br>2-No<br>9-N/A	
2.69	Did you face any difficulties in the toilet?<br>1-Yes<br>2-No<br>9-N/A	

2.70	If yes, then what type of problem you have faced?<br>(Multiple answer allowed)<br>1-Mentioned<br>2-Not mentioned	
	a. Insufficient space in the toilet area for wheelchair user	
	b. No handrail	
	c. Slippery floor	
	d. No facility for wheelchair user	
	e. Insufficient lighting	
	f. Mirror is not an appropriate angled	
	g. Rubbish bin is not accessible	
	h. Basin is not an appropriate angled	
	i. Other-Specify<br>____	
2.71	Have you found the toilet accessible?<br><br>5-Very accessible 4- accessible 3-Neutral 2- inaccessible 1-Very inaccessible	
	**Maternity Services(women with disabilities)**	
2.72<br>**If not yes go to 3.1**	Did you take any maternal service from a facility?<br>1-Yes<br>2-No<br>9-N/A	
2.73	Did you find any adjustable bed for the women with the disabilities?<br>1-Yes<br>2-No<br>9-N/A	
2.74	Did you find any weight scale available for wheelchair user?<br>1-Yes<br>2-No<br>9-N/A	
2.75	Did you find any adjustable baby cots/incubator for wheelchair user?<br>1-Yes<br>2-No<br>9-N/A	
2.76	Did you find any assistance for your activities of daily life from the facility?<br>1-Yes<br>2-No<br>9-N/A	
2.77	Have you been explained all necessary information regarding ANC, PNC?<br>1-Yes<br>2-No<br>9-N/A	
2.78	How satisfied are you to get maternity service in a facility?<br><br>5-Very satisfied 4-Satisfied 3-Neutral 2-Dissatisfied 1-Very dissatisfied	

3. Suggestions		
3.1	Information- What can be done to get the accessible information in a facility?<br>(Multiple answer allowed)<br>1-Mentioned<br>2-Not mentioned	
	a. Assigned trained hospital Staff	
	b. Braille imprinting	
	c. Booklet	
	d. Using Sign	
	e. Pictorial	
	f. Other-Specify<br>____	
3.2	Communication- What can be done to communicate with PWDs in a facility effectively?<br>(Multiple answer allowed)<br>1-Mentioned<br>2-Not mentioned	
	a. Assigned trained hospital Staff	
	b. Sign language interpreter	
	c. Braille imprinting	
	d. Booklet	
	e. Using Sign	
	f. Pictorial	
	g. Hearing loop	
	h. Other-Specify<br>____	
3.3	Infrastructure- What can be done to improve the infrastructure for PWDs in a facility?<br>(Multiple answer allowed)<br>Mentioned-1<br>Not mentioned-2	
	a. Obstruction free entrance	
	b. Handrail where needed	
	c. Accessible elevator	
	d. Clear sign/symbols	
	e. Tactile floor	
	f. Ramp	
	g. Available wheelchair	
	h. Toilet (accessible toilet for PWDs)	
	i. Other-Specify<br>____	

3.4	Capacity Development of Health Service Provider- What can be done to capacity development of health service provider for PWDs in a facility?<br>(Multiple answer allowed)<br>1-Mentioned<br>2-Not mentioned	
	a. Awareness training	
	b. Tailored specific training for different disabilities	
	c. Other, specify<br>_______________________________________	
3.5	How MNCAH services could be made disability friendly? (For women with disabilities)<br>(Multiple answer allowed)<br>1-Mentioned<br>2-Not mentioned<br>9- N/A	
	a. By prioritizing to get the service	
	b. By making a counseling corners for the PWDs	
	c. By making a specific service room with accessible furniture and equipment for the PWDs for each MNC & AH services	
	d. By giving special services such as, home visit	
	e. Others , please specify<br>____	

Form - 08

2018 Observation checklist for health facility assessment

Mainstreaming Disability Friendly Health Services (DFHS) focusing MNC&AH program

Centre for injury prevention and research, Bangladesh (CIPRB)

Form 8- Checklist for disability friendly infrastructure and services

Name of research assistant-

Date of data collection-

**dd**	**mm**	**yy**

Type of survey location	Name	Code	Time of interview
District		☐☐	**Start time of interview-** ☐☐ : ☐☐
Upazila		☐	
Union		☐	
Health facility (please tick the health facility and write the code)	Specialized hospital	☐☐	**End time of interview-** ☐☐ : ☐☐
	Medical college hospital		
	District hospital		
	Maternal & child welfare centre		
	Upazila health complex		
	Union health & family welfare centre / Union health centre/ Union sub-centre		
	Community clinic		
Unique ID - ☐☐☐☐☐☐			

Form - 08

2018 Observation checklist for health facility assessment

Sl #	A checklist for disability friendly infrastructure and services	1-YES<br>2- No<br>9- N/A
**1**	**General points**	
1.1	Can people with disabilities come into the facility (enough space for vehicle) easily?	
1.2	Can the facility vacant (enough space) people with disabilities safely in an emergency?	
1.3	Does the facility have wheelchairs where people need them?	
1.4	Does the furniture and equipment of the facility is suitable in terms of height, reach for people with disabilities?	
**2**	**Entrance, foyer, exit and parking area**	
2.1	Does the entrance of the facility free from any obstructions?	
2.2	Does the facility have accessible pathways to the building?<br>- step-free entrance or<br>- both steps and a gentle ramp pathways with continuous handrails	
2.3	Does the entrance are provided with tactile floor for blind people?	
2.4	Does the facility have the entry and exit point that is clearly sign-posted near the entrance?	
2.5	Does before step have any continuous handrails?	
2.6	Does before step have any braille inscription (blueprint of the architecture of the facility)?	
2.7	Does the entrance doors easy to open or do they open automatically?<br>**If 2.7 is yes then go to 2.11**	
2.8	Are there pull/lock handles to open/close the door if there is no self-locking device?	
2.9	Are door pull/lock handles visible and at a height (at least 80 cm) which a wheelchair user can access?	
2.10	If there is a double set of doors at an entrance, is there minimum 100 cm for moving wheelchair between the outer and inner doors for someone with limited mobility or a wheelchair user?	
2.11	Is there accessible parking space near to the entrance?	
2.12	Does the parking area of the facility free from any obstruction?	
2.13	Does the parking area marked with universal symbol of disability?	
**3**	**Staircase (Upazila Health Complex and higher level hospitals)**	
3.1	Are there continuous handrails provided on either side of the staircase and positioned between 85 cm and 100 cm above floor finish? **(if No, go to 3.3)**	
3.2	Is the handrail extending a minimum of 30 cm beyond the top and bottom steps, turning to the wall?	
3.3	Is there braille inscription at the handrail?	
3.4	Is there contrasted texture (floor roughness) in the floor that indicates that stairs are ahead for visual disability?	

2018 Observation checklist for health facility assessment

3.5	Are the steps non-slippery?	
3.6	Are the steps marked along the edges?	
3.7	Is there a landing space at the top and bottom of a staircase?	
3.8	Is steps are uniform (height - not higher than 15cm and width- not less than 30cm)?	
**4**	**Ramp(where applicable)**	
4.1	Is the ramp free from any obstructions?	
4.2	Is the ramp anti-slippery?	
4.3	Is the ramp at least (120-200) cm wide?	
4.4	Are ramps no steeper than 1:12-1:20?	
4.5	Are ramps no longer than 100 cm?	
4.6	Is there handrails provided on both sides?	
4.7	Is step ramp provided wherever an accessible route crosses a step?	
4.8	Does the tactile floor locate at least 40 cm before the ramp?	
**5**	**Reception and waiting areas**	
5.1	Is there clear signage showing at a height of 130 cm- 160 cm above floor level where to find different services and facilities?	
5.2	Is the reception desk close to the entrance?	
5.3	Is there a two-tier height reception desk that can serve both those who are standing and those who are seated (including wheelchair users) with adequate knee space?	
5.4	Is there available chair (presence of chair for all PWDs or caregiver except wheelchair user) at the reception desk for the person making inquiries?	
5.5	Is there available wheelchair at the reception desk for the wheelchair user?	
5.6	Is there a loop system at reception desks to facilitate those with hearing aids?	
5.7	Is there good lighting on the receptionist's face to facilitate lip-reading?(the room with sufficient light that can be access of sunlight in case of daytime)	
5.8	Is there adequate space (150×150 cm to 180×180 cm) for both manual and powered wheelchairs to enter and turn around in the reception area?	
5.9	Is there adequate seating with armrest in any waiting area?	
5.10	Is there a sign of international accessibility for PWDs in the reception if there is separate reception desk for PWDs?	
5.11	Can people with limited handiness take a leaflet from a leaflet display stand easily?(height of 130 cm -160 cm from floor level)	
**6**	**General areas and circulation**	
6.1	Are there non-slip floor surfaces that are dry, well-maintained and easy to use by someone with a walking aid or in a wheelchair?	
6.2	Are there contrasts in color for floor coverings to direct different areas of the building?	
6.3	Are there contrasts in texture for floor coverings to direct different areas of the building?	

2018 Observation checklist for health facility assessment

6.4	Are doorways, corridors and circulation spaces wide enough for powered or manual wheelchair users to navigate and turn?	
6.5	Are corridors/ waiting rooms free of any obstacles that could restrict mobility or cause injury?	
6.6	Are there handrails with seating in all waiting areas or along circulation routes to enable a person with a physical difficulty or balance difficulties to get around the building and take a break if they need it?	
6.7	Are there handrails where there are changes in floor levels and where there are steps or ramps?	
6.8	Is there a glazed vision panels on doors so that one can see what is beyond the door?	
6.9	Are there markings on glass doors so that they can be clearly seen?	
6.10	Is there an alternative for those who cannot use steps, such as a lift or a ramp at different floor level?	
**7**	**Toilet facilities**	
7.1	Is door space (at least 90 cm) of toilet facilities accessible/wide?	
7.2	Is the toilet area wide/big enough for wheelchair user (20 cm× 170 cm including a hand basin and turning circle)?	
7.3	Is there an accessible toilet near the examination room so that a person can give a urine specimen, if required?	
7.4	Are the door handles (lock handle) should be usable by people with restricted handiness (e.g. operable with an elbow or one hand)?	
7.5	Are the toilet taps (handle) should be usable by people with restricted handiness?	
7.6	Are there appropriately placed grab rails (30 cm from back of toilet) ?	
7.7	Does handrails provided at 80 cm high from the floor and adjustable?	
7.8	Does the lock of the door at 80 cm high from the floor that the locks can be locked for wheelchair user?	
7.9	Is the floor non-slippery?	
7.10	Are there toilet with handrail/ special design with hole inside the toilet for PWDs?	
7.11	Are accessible toilets maintained and repaired promptly if out of order?	
7.12	Are there international accessible symbol for PWDs in the toilet?	
7.13	Does the toilet have a chair with monkey pole if there is no commode inside the toilet?	
**8**	**Consulting rooms**	
8.1	Are the doors of consulting room wide (at least 90cm) enough to allow wheelchairs?	
8.2	Does consulting rooms have sufficient space (at least 150 cm^2) to enable a manual or powered wheelchair user to turn?	
8.3	Can the beds of consulting room accommodate a lifting pull to enable a person to transfer safely and comfortably onto an examination or treatment table or chair?	

2018 Observation checklist for health facility assessment

8.4	Are examination chairs centrally located with access from both sides (or can they be moved easily so that they are)?	
8.5	Are there accessible weight machines so that people who are not able to stand on the weight scale can be weighed as for wheelchair user?	
8.6	Are handgrips provided to help people with mobility or vision impairments to have support when standing beside an on weight scales?	
8.7	Is there any trained/assigned doctor or health service provider on sign language, for collecting history from person with speech and hearing disability?	
	**Pathology room (UHC and higher level hospitals)**	
8.8	Are the doors of pathology room wide (at least 90cm) enough to allow wheelchairs?	
8.9	Does pathology room have sufficient space (at least 150 cm^2) to enable a manual or powered wheelchair user to turn?	
8.10	Are there available wheelchairs/accessible chair for collecting sample?	
8.11	Is there accessible toilet for the PWDs near the pathology room to collect specimen?	
8.12	Is there any trained/assigned health service provider on sign language, for communicating with person with speech and hearing disability?	
	**Radiology/Imaging room (UHC and higher level hospitals)**	
8.13	Are the doors/entrance of radiology/imaging room wide (at least 90cm) enough to allow wheelchairs?	
8.14	Does radiology/imaging room have sufficient space (at least 150 cm^2) to enable a manual or powered wheelchair user to turn?	
8.15	Are diagnostic equipment’s such as mammography machine/X-ray/CT scan/ultrasound capable of being accessed by a PWD?	
8.16	Are handgrips provided to help people with mobility or vision impairments to have support when standing beside a diagnostic machine?	
8.17	Is there any trained/assigned health service provider on sign language, for communicating with person with speech and hearing disability?	
	**Procedure room**	
8.18	Are the doors of procedure room (IUD/DNC/MR/dressing/suture) wide (at least 90cm) enough to allow wheelchairs?	
8.19	Does procedure room have sufficient space (at least 150 cm^2) to enable a manual or powered wheelchair user to turn?	
8.20	Can the beds of procedure room accommodate a lifting pull to enable a person to transfer safely and comfortably onto an examination or treatment table or chair?	
8.21	Are examination chairs centrally located with access from both sides (or can they be moved easily so that they are)?	
8.22	Are there accessible weight machines so that people who are not able to stand on the weight scale can be weighed as for wheelchair user?	
8.23	Are handgrips provided to help people with mobility or vision impairments to have	

	support when standing beside an on weight scales?	
8.24	Is there any trained/assigned doctor or health service provider on sign language for communicating with person with speech and hearing disability?	
**9**	**Hospital wards/inpatient department (UHC and higher level hospitals)**	
9.1	Does the entrance of hospital ward accessible (at least 90 cm wide) to PWDs?	
9.2	Is there sufficient space (at least 150 cm^2) around a bed for a manual and powered wheelchair user to turn beside a bed in a hospital ward?	
9.3	Are there accessible and automatic height-adjustable beds available if service user/PWDs need one?	
9.4	Are lifters available to enable people be lifted or to lift themselves, in particular to enable transfer between bed and bathroom or into and out of a bedside chair?	
9.5	Is there sufficient space around a bed for a manual and powered wheelchair user to turn beside a bed?	
9.6	Is there a fully accessible toilet (that mentioned earlier) adjacent to the ward?	
9.7	Is there a single room available on all wards, which can help offer more privacy or quiet for those who require it because of their illness or disability, where possible? Some disabilities may result in a person being uncomfortable or find it difficult to communicate in a noisy or bright environment.	
9.8	Is there a range of chairs with arm rests in the ward to suit people with limited mobility and to assist them when they go to stand?	
9.9	Is the environment free of obstacles or hazards (Minimum height clearance of corridors e.g. 220 cm)?	
9.10	Is there a television provided?	
9.11	If yes, is the option of subtitles available?	
10	**Out-patient department (UHC and higher level hospitals)**	
10.1	Is the out-patient department/specific service area obstacle free?	
10.2	Is there a handrail in the out-patient/service room to assist people who are not steady on their feet?	
10.3	Is the entrance accessible/wide enough (at least 90 cm) for the wheelchair user?	
10.4	Can the beds of room accommodate a lifting pull to enable a person to transfer safely and comfortably onto an examination or treatment table or chair?	
10.5	Are examination chairs centrally located with access from both sides (or can they be moved easily so that they are)?	
10.6	Are there accessible weight machines so that people who are not able to stand on the weight scale can be weighed as for wheelchair user?	
10.7	Are handgrips provided to help people with mobility or vision impairments to have support when standing beside an on weight scales?	
10.8	Is there a hearing loop?	
10.9	If so, are people who use hearing aids routinely informed that it is available?	
10.10	Is there designated hospital staff for information and communication with people with	

	disabilities may have?	
10.11	Do people with intellectual disabilities in the facility access primary health care?	
10.12	Are people with disabilities actively included in preventive and screening programs?	
10.13	Does the facility have a register of patients with disabilities to enable audit of their care?	
10.14	Is there an alternative for patients who have difficulties in negotiating stairs; where lifts are not provided, for example, that they can be seen in a downstairs consulting room?	
10.15	Is there an accessible toilet located at the specific service area (OPD, Primary health care room)?	
**11**	**Signs and notices**	
11.1	Is there a clear way-finding system around the building?(e.g.- colour coding)	
11.2	Is there clear signage at an appropriate height (130cm-160cm) above floor level?	
11.3	Are the signs made from a material that does not reflect light?	
11.4	Is plain language used in signs and notices?	
11.5	Do the signs use clear and consistent language and images?	
11.6	Is there a color contrast between lettering and background on information notices and signs?	
11.7	Are picture signs and symbols used that can be readily understood by all; for example, people with literacy difficulties, people with intellectual disabilities and people who do not read?	
11.8	Is there a notice where you have a hearing loop?	
11.9	Are notices in a minimum of 18pt font?	
11.10	Do they use large symbols with a combination of upper and lower case lettering?	
11.11	Do the facility use any sign policy/guideline?	
11.12	Do the facility using the international symbol of accessibility for the people with disabilities where needed?(e.g. – accessible toilet, reception, medicine corner, room with symbol for the PWDs)	
11.13	Are written notices in large print, in a clear typeface, with good color contrast and on a matt background to reduce shine?	
**12**	**Safe evacuation (where applicable- UHC and upper level hospitals)**	
12.1	Is there an appropriate plan in place for the safe evacuation of everyone, including people with disabilities, in the case of an emergency?	
12.2	Does the facility have any audio warning system for persons with visual disability?	

Form - 08

2018 Observation checklist for health facility assessment

12.3	Does the facility have flash light warning system for deaf people who cannot hear any alarms?	
**13**	**Elevators (If applicable- UHC and upper level hospitals)**	
13.1	Is the door entrance more than 90cm wide?	
13.2	Is the button accessible for wheelchair user (In terms of height button should be placed at 85-120 cm height) to assist a child or someone using wheelchair?	
13.3	Is the elevator space (110 cm×140 cm) accessible for wheelchair user?	
13.4	Is the Braille code facility available for visually impaired people?	
13.5	Are the voice projection/ talking lift facility available for visually impaired people?	
13.6	Is a Handrail provided in the elevator (Handrails should be installed mounted 80 cm to 85 cm from the floor)?	
**14**	**Maternity service**	
14.1	Is the door entrance of ANC/PNC room more than 90cm wide?	
14.2	Is the delivery room entrance more than 90cm wide?	
14.3	Is there accessible/adjustable labour bed for the women with disabilities?	
14.4	Does maternity ward has sufficient space (at least 150 cm^2) to enable a manual or powered wheelchair user to turn?	
14.5	Are there accessible and automatic height-adjustable beds available if service user/women with disabilities need one?	
14.6	Are lifters available to enable pregnant women with disabilities be lifted or to lift themselves, in particular to enable transfer between bed and bathroom or into and out of a bedside chair?	
14.7	Does the height adjustable baby cots/incubators for wheelchair user?	
14.8	Is there a fully accessible toilet (that mentioned earlier) adjacent to the ward?	
14.9	Does the weight scale is available for wheelchair user?	
14.10	Are handgrips provided to help women with disabilities (mobility or vision impairments) to have support when standing beside an on weight scales?	
14.11	Is there any trained/assigned health service provider on sign language, for communicating with women with speech and hearing disability?	
14.12	Is the accessible toilet available near the delivery room/ward?	
**15**	**Rehabilitation (District hospital and higher level facilities)**	
15.1	Does the facility have physiotherapy rehabilitation center for restricted mobility/handiness?	
15.2	Does the facility have occupational therapy rehabilitation center?	
15.3	Does the facility have speech & language therapy rehabilitation center?	
**16**	**Capacity of the Health service providers**	
16.1	Does the facility have trained/designated staff to provide information for the people with the disabilities?	
16.2	Does the facility have trained/designated staff to communicate with people with the disabilities?	

2018 Observation checklist for health facility assessment

Form - 08

**17**	**Medicine corner/Pharmacy**	
17.1	Does the facility have a medicine corner that is available at a height (130 cm-160 cm) that is appropriate for people with disabilities?	
17.2	Is the medicine corner for the people with the disabilities using international sign for accessibility?	
**18**	**Ticket counter**	
18.1	Does the facility have a ticket counter that is available at a height (130 cm-160 cm) that is appropriate for people with disabilities?	
18.2	Is the ticket counter for the people with the disabilities using international sign for accessibility?	

Mainstreaming Disability Friendly Health Services (DFHS) focusing MNC&AH program

Centre for injury prevention and research, Bangladesh (CIPRB)

Form 9- Structure Questionnaire-Health facilities administrators

Name of research assistant-

Date of data collection-

**dd**	**mm**	**yy**

Type of Survey Location	Name	Code	Time of interview
District		☐☐	**Start time of interview-** ☐☐ : ☐☐
Upazila		☐	
Union		☐	
Health facility (please tick the health facility and write the code)	Specialized Hospital	☐☐	**End time of interview-** ☐☐ : ☐☐
	Medical college hospital		
	District hospital		
	Maternal & child welfare centre		
	Upazila health complex		
	Union health & family welfare centre / Union health centre/ Union sub-centre		
	Community clinic		
Health facility administrator		☐	
Designation			
Unique ID - ☐☐☐☐☐☐☐			

Structure questionnaires for the health facilities administrators’

Form - 09

Questionnaires	Code
1. Perception on Disability Friendly Health Services	
1.1Do the facility have services for People With Disabilities (PWDs)?<br>1-Yes<br>2-No	
1.2 Do PWDs come to this facility looking for services?<br>1-Yes<br>2-No	
1.3 How many PWDs on an average per month do you treat?<br><br>____	
1.4 How many types of PWDs come to get services in your facility?<br>(Multiple answer allowed)<br>1-Mentioned<br>2-Not mentioned	
Types of disabilities – (According to Disabilities Rights and Protection Act 2013)	
a. Autism/ Autism Spectrum Disorder	
b. Physical	
c. Psychosocial /Mental	
d. Visual	
e. Speech	
f. Intellectual	
g. Hearing	
h. Deaf Blind	
i. Cerebral Palsy	
j. Down Syndrome	
k. Multiple Disability	
l. Other, specify<br><br>____________________________________	
1.5 Do you know about any Laws/Acts/Sector wise program of our country on disability regarding health facilities/services?<br>1-Yes<br>2-No	

1.6 If yes, can you mention the title of the Acts/Laws/Policies/Sector wise program? (Multiple answer allowed)<br><br>1-Mentioned<br>2-Not mentioned	
a. National Health Policy 2011	
b. Disability Welfare Act 2001	
c. National strategy for Autism and NDDs	
d. 4th HPNSP 2017-2021	
e. Disabilities Rights and Protection Acts 2013	
f. Other-Specify ________________________________	
1.7 Is there any Disability Friendly Policy in place/displayed in your facility?<br>1- Yes<br>2-No	
1.8 If yes, what are the policies? Please specify(multiple answer allowed)<br><br>1-Mentioned<br>2-Not mentioned	
a. Autism friendly	
b. Neurodevelopment Disorders (NDDs) friendly	
c. Braille	
d. Sign language interpreter	
e. Wheelchairs	
f. Hearing aid	
g. Getting health services on priority based	
h. Free health service for PWDs	
i. Others-specify ________________________________	
**2. Situation assessment in terms of DFHS**	
**Accessible Information**	
2.1 How does the facility assisted to give information for the PWDs? (multiple answer allowed)<br><br>1-Mentioned<br>2-Not mentioned	
a. using any booklet for service information	
b. using braille	
c. using any hearing device	

d. using pictorial	
e. using external or internal sign for direction	
f. caregiver	
g. hospital staff assistance	
h. other specify ____	
**Accessible Communication**	
2.2 How does the facility assisted to communicate with the people with the disabilities? (Multiple answer allowed)<br>1-Mentioned<br>2-Not mentioned	
a. facility staff use sign language to communicate	
b. facility staff communicate through braille	
c. facility have hearing loop facility	
d. noise free room designed to communicate with intellectual, autism, mental & cerebral palsy PWD's	
e. Through caregiver	
f. other(Please specify) ____	
**Accessible Infrastructure**	
2.3 In which part PWD's faced challenges in the infrastructure of this facility? (Multiple answer allowed)<br>1-Mentioned<br>2-Not mentioned	
a. Entrance	
b. Reception	
c. General areas and circulation	
d. Toilet facilities	
e. Consulting and treatment rooms	
f. Hospital wards	
g. Signs and notices	
h. Elevator	
i. Safe evacuation /Emergency exit	
j. Medicine corner	
k. Other- Specify ____	

Structure questionnaires for the health facilities administrators’

Form - 09

**Capacity building of the health service provider**	
2.4 Is there any staff assigned to provide information to the PWDs?<br>1-Yes<br>2-No	
2.5 Is there any staff assigned to communicate with the PWDs?<br>1-Yes<br>2-No	
2.6 Do you think staff of this facility is trained to provide information considering all disabilities?<br>1-Yes<br>2-No	
2.7 Do you think staff of this facility is trained to communicate with the PWD’s?<br>1-Yes<br>2-No	
2.8 Is any of the hospital staff got awareness training on disabilities?<br>1-Yes<br>2-No	
**Transport**	
2.9 Does the facility give transport service to the PWDs?<br>1- Yes<br>2-No<br>9-Not applicable	
3. Challenges on DFHS	
3.1 What will be your challenge to make your facility a disability friendly?<br>(Multiple answer allowed)<br>1-Mentioned<br>2-Not mentioned	
a. Information	
b. Communication	
c. Infrastructure	
d. Capacity building of the health service provider	
3.2 If Information- Please specify (multiple answer allowed)<br>1- mentioned<br>2- not mentioned	
a. Costly to provide sign/symbol everywhere	
b. No sufficient space	
c. Not aware what information to be provided	
d. Need to give training to the hospital staff how to provide information to the PWDs	
e. Don’t know anything about accessible information for the PWDs	

f. Other, specify ______________________	
3.3 If Communication – Please specify (multiple answer allowed)<br>1-mentioned<br>2-not mentioned	
a. Costly to provide braille	
b. Need to train hospital staff on sign language	
c. Not affordable to provide hearing loop	
d. Not affordable to provide pictorial	
e. Not affordable to provide audio	
f. Not affordable to provide video	
g. Not availability of the communicative devices	
h. Don't know anything about effective communication for the PWDs	
i. Other, specify ______________________	
3.4 If Infrastructure-please specify(multiple answer allowed)<br>1- mentioned<br>2-not mentioned	
a. Not feasible to improve all infrastructure part	
b. Not affordable	
c. Not applicable in my facility	
d. No space to restructure	
e. Not possible locally	
f. Other, specify ______________________	
3.5 If Capacity developing of the service provider- please specify<br>(multiple answer allowed)<br>1-mentioned<br>2-not mentioned	
a. Insufficient health service provider to take responsibility	
b. Health service provider will not be interested to take training	
c. Not affordable	
d. No such initiative taken by DGHS	
e. Not aware of such training	
f. Not applicable in this facility	
g. Other, specify ______________________	

Structure questionnaires for the health facilities administrators'

Form - 09

4. Suggestions to improve the situation	
4.1 In which domain the facility need to be improved to make disability friendly?<br>(multiple answer allowed)<br>1-Mentioned<br>2-Not mentioned	
a. Information	
b. Communication	
c. Infrastructure	
d. Capacity developing of the service provider	
e. Other, specify____	
4.2 Information- What can be done to get the accessible information for the PWDs in a facility?<br>(Multiple answer allowed)<br>1-Mentioned<br>2-Not mentioned	
a. By giving training to the hospital staff for accessible information	
b. Providing braille imprinting	
c. Providing booklet	
d. Using sign	
e. Using Pictorial/signboard	
f. Other, specify<br>____	
4.3 Communication- What can be done to improve communication with PWDs in a facility?<br>(Multiple answer allowed)<br>1-Mentioned<br>2-Not mentioned	
a. By giving training to the hospital Staff for communication with PWDs	
b. By recruiting sign language interpreter	
c. By braille imprinting	
d. Providing booklet	
e. Using Sign	
f. Using Pictorial	
g. Providing hearing loop	
h. Giving training to the PWDs on the sign language	
i. Giving training to the PWDs on the braille	
j. Other-Specify<br>____	

Structure questionnaires for the health facilities administrators’

Form - 09

4.4 Infrastructure-What can be done to improve the infrastructure for PWDs in a facility?<br>(Multiple answer allowed)<br>1-Mentioned<br>2-Not mentioned	
a. Making obstruction free entrance	
b. Providing handrail where needed	
c. Providing accessible elevator	
e. Using clear sign/symbols	
f. Providing tactile floor	
g. Providing ramp	
h. Providing available wheelchair	
i. Providing toilet (accessible toilet for PWDs)	
j. Other-Specify<br>____	
4.5 Capacity developing of the Health service provider- What can be done to capacity development of health service provider for PWDs in a facility?<br>(Multiple answer allowed)<br>1-Mentioned<br>2-Not mentioned	
a. Awareness training	
b. Tailored specific training	
c. Others, specify________________________________	
**Maternal Neonatal Child and Adolescent Health (MNCAH) services**	
4.6 Does the facility give MNCAH services?<br>1 -Yes<br>2 -No<br>9 - N/A	
4.7 How MNCAH services could be made disability friendly?<br>(Multiple answer allowed)<br>1-Mentioned<br>2-Not mentioned	
a. By prioritizing to get the service	
b. By making a counseling corners for the PWDs for each services	
c. By making a specific service room with accessible furniture and equipment for the PWDs	
d. By giving special services such as, home visit	
e. By giving training to the hospital staff related to MNCAH services	
f. Other, specify<br>____	
